# Optimal resource allocation in HIV self-testing secondary distribution among Chinese MSM: data-driven integer programming models

**DOI:** 10.1098/rsta.2021.0128

**Published:** 2022-01-10

**Authors:** Fengshi Jing, Qingpeng Zhang, Jason J. Ong, Yewei Xie, Yuxin Ni, Mengyuan Cheng, Shanzi Huang, Yi Zhou, Weiming Tang

**Affiliations:** ^1^ Institute for Healthcare Artificial Intelligence, Guangdong Second Provincial General Hospital, Guangzhou 510317, People’s Republic of China; ^2^ University of North Carolina Project-China, Guangzhou, People’s Republic of China; ^3^ School of Data Science, City University of Hong Kong, Hong Kong SAR, People’s Republic of China; ^4^ Faculty of Infectious and Tropical Diseases, London School of Hygiene and Tropical Medicine, London, UK; ^5^ Central Clinical School, Monash University, Melbourne, Australia; ^6^ Duke Global Health Institute, Duke University, Durham, NC, USA; ^7^ Zhuhai Center for Diseases Control and Prevention, Zhuhai, People's Republic of China; ^8^ Faculty of Medicine, Macau University of Science and Technology, Macau SAR, People’s Republic of China; ^9^ Division of Infectious Diseases, Department of Medicine, UNC School of Medicine, University of North Carolina at Chapel Hill, Chapel Hill, NC, USA

**Keywords:** HIV self-testing, secondary distribution, integer programming, greedy algorithm, mathematical optimization

## Abstract

Human immunodeficiency virus self-testing (HIVST) is an innovative and effective strategy important to the expansion of HIV testing coverage. Several innovative implementations of HIVST have been developed and piloted among some HIV high-risk populations like men who have sex with men (MSM) to meet the global testing target. One innovative strategy is the secondary distribution of HIVST, in which individuals (defined as indexes) were given multiple testing kits for both self-use (i.e.self-testing) and distribution to other people in their MSM social network (defined as alters). Studies about secondary HIVST distribution have mainly concentrated on developing new intervention approaches to further increase the effectiveness of this relatively new strategy from the perspective of traditional public health discipline. There are many points of HIVST secondary distribution in which mathematical modelling can play an important role. In this study, we considered secondary HIVST kits distribution in a resource-constrained situation and proposed two data-driven integer linear programming models to maximize the overall economic benefits of secondary HIVST kits distribution based on our present implementation data from Chinese MSM. The objective function took expansion of normal alters and detection of positive and newly-tested ‘alters’ into account. Based on solutions from solvers, we developed greedy algorithms to find final solutions for our linear programming models. Results showed that our proposed data-driven approach could improve the total health economic benefit of HIVST secondary distribution.

This article is part of the theme issue ‘Data science approaches to infectious disease surveillance’.

## 1. Introduction

Men who have sex with men (MSM) are currently the most vulnerable community populations affected by the human immunodeficiency virus (HIV). In China, the percentage of MSM recorded as new HIV infection cases is still rising [1]. HIV status unawareness is the main factor leading to the current HIV epidemic among MSM. Providing routine testing to enable infected individuals to know their HIV-positive status and facilitating their treatment initiation contributes significantly to HIV prevention interventions [2]. Over 40% of Chinese MSM have never been tested [3], and over 30% of HIV-infected individuals remain unaware of their serostatus [4]. An innovative and effective strategy named HIV self-testing (HIVST) that can improve the protection of the testers’ privacy and increase their willingness to test has been recommended by the World Health Organization (WHO) for global consideration [5,6]. HIVST is an optimal strategy to address the persisting concerns about HIV testing like stigma and the lack of trust towards medical institutions [7] and to reach marginalized populations at high risk of infection.

Drawing on the progress made by HIVST, secondary distribution further expands HIV testing coverage. It has been developed and piloted in many countries recently [8–12]. In the secondary distribution of HIVST among MSM, individuals (defined as indexes) obtain several HIVST kits and then distribute these testing kits to members within their social networks like stable sexual partners, casual sex partners and MSM community friends (defined as ‘alters’). Our previous work has implemented a secondary distribution program of HIVST from September 2019 to September 2020 in China [11,12], which demonstrated the effectiveness of HIVST secondary distribution in expanding testing coverage.

As an emerging form of distribution, current research into secondary HIVST distribution mainly focuses on the development and validation of intervention approaches (e.g. using online social media platforms [10], employing monetary and psychological incentives [11,12]) to better promote the secondary distribution of HIVST, and verify these through traditional public health methodologies such as a randomized controlled trial (RCT) [11] or a quasi-experiment [13]. Nonetheless, there is still much room for secondary HIVST distribution optimization where mathematical modelling could make a difference. Usually, the same number of kits indexes applied for will be dispatched to all indexes for further distribution to alters in this current model. However, a waste of undistributed kits usually happens as some indexes fail to find suitable alters willing to use them. For example, in our previous study, the 309 indexes applied for 759 kits for distribution. Unfortunately, they only distributed 269 kits (i.e. successfully found 269 unique alters), and over 60% (490 test kits) were wasted [11,12]. In a developed or developing country, resources for HIV testing kits might be rich enough. However, the cost of self-testing is relatively high in low-income and least-developed country where healthcare resources are always constrained [14]. Hence, such waste of self-testing kits could impact the further implementation of secondary distribution of HIVST.

Considering a resource-constrained situation of testing kits in the secondary distribution of HIVST, this paper proposed two original data-driven integer programming models. We used mathematical models to determine the number of test kits dispatched to each index participant instead of self-application to achieve optimal resource distribution. In our integer programming models, the objective function addressed the overall health economic benefits of HIVST secondary distribution based on our actual implemented trial among Chinese MSM [11,12], where the economic benefits of covering regular alters and positive & newly tested alters were all taken into consideration. We developed greedy algorithms to obtain final solutions to the linear programming models based on the solver’s solutions. The final results showed that the proposed data-driven approach could enhance the health economic benefits of HIVST secondary distribution.

The other aspects of this study are as follows: §2 reviewed some related work. Section 3 illustrates these two linear-integer programming models derived from our actual data. The following §4 explains our greedy algorithms and §5 describes the results. Finally, we present the conclusions in §6.

## 2. Literature review

Our paper relates to existing literature on resource-allocation problems for HIV prevention and control. Most previous studies address such problems from a macroscopic view, considering the global optimal resource allocation for HIV prevention and treatment instead of a specific HIV prevention/control program. For example, Zaric & Brandeau [15] built a mathematical optimization model to determine how to optimally allocate HIV prevention funds for many HIV prevention measures (i.e. how to allocate funds for each HIV prevention program) and evaluated healthcare outcomes based on different optimization methods [15]. And Kaplan & Merson [16] discussed the trade-off between efficiency and equity when allocating HIV prevention resources [16]. Additionally, Brandeau *et al.* [17] adopted information relevant to HIV prevention program production functions with similar limited-resource settings to determine how many resources to dispatch to each HIV prevention program [17]. Lasry *et al.* [18,19] proposed an optimization model with linear constraints for allocating HIV prevention resources for the Centers for Disease Control and Prevention (CDC) of the United States [18]. The model showed a good outcome for public health if their model could influence the CDC’s decision [19]. Furthermore, Alistar *et al.* [20] studied the problem of allocating limited resources between HIV prevention and treatment. The objective function of that study considered the reproduction number $R_{0}$, and thus, they aimed to minimize the function of $R_{0}$ [20]. This work targeted the overall resource allocation for two points in the HIV control process (i.e. prevention & treatment). Furthermore, Deo *et al.* [21] optimized global resource allocation for three aspects of HIV control at the Veterans Health Administration, including HIV screening, HIV testing and HIV care [21].

On the other hand, few aspects of studying limited resource-allocation problems in HIV control are concentrated on a specific strategy/program from a micro point of view. For instance, to better control infant HIV-infection, Deo & Sohoni [22] showed how to allocate point-of-care devices in resource-limited settings [22], and Jónasson *et al.* [23] described optimal locations for diagnostic equipment and laboratories [23]. Both strategies led to the efficiency enhancement of early infant diagnosis of HIV. The studies referenced above share similar motivation with this present study as they all consider allocating a fixed budget or resource for the optimal control of HIV prevention. However, our work is different as it examines a very unconventional problem with distinct dynamics (i.e. secondary distribution of HIVST).

## 3. Integer programming models

We based our data-driven integer programming models on our two previous studies [12,24]. We conducted an HIVST secondary distribution program among Chinese MSM, and there were 309 indexes with 269 alters. Some of the alters had newly tested, while some were HIV-positive testers. It’s meaningful for an index to distribute an HIVST kit to a new tester as previous studies showed that over 40% of Chinese MSM have never been tested [3]. Increasing HIV testing yield and identifying more HIV-infected alters unaware of their status played a vital role in HIV infection control. Thus, it could be more crucial to detect a newly tested alter or an HIV-infected alter. We estimated the economic value and benefit of distributing the HIVST kit to an alter and the alter being a new tester or HIV-positive from a health economics perspective in our previous study [12].

### (a) Model I

In Model I, we only consider allocating HIV testing kits to key influential indexes. Indexes not identified as significant influencers by the ensemble machine learning model [24] were therefore not considered. Hence, we only concentrated on key influencers like factors that optimize the secondary distribution of HIVST kits. In practice, this stream releases the pressure of healthcare service providers or social workers as they only need to work for around 20% key influential indexes with full attention.

The number of kits to be allocated to each essential influential index was determined using Model I. That was with the final goal of maximizing the economic benefit, which considers the overall effect of normal alters expansion (i.e. distributing one kit to an alter for HIV testing) and positive & newly testing alters detection (i.e. this alter is confirmed later as HIV-positive case or first-time tester). We define the sets, parameters, and variables in the following table 1. These above settings are data-driven from our actual implementation of an HIVST secondary distribution program, and thus our model is data-driven in a sense. Then the Model I is defined as followed.

**Table 1.** Model I notations.

*sets*	
K	set of key influential indexes
A	set of types of alters
parameters	
k	indices of key influential indexes
a	indices of types of alters
${\text{Pr}}^{a,k}$	probability of key influential index k distributing an HIVST kit to an alter of type a
$E^{a}$	economic benefit of distributing an HIVST kit to an alter of type a
$S_{k}$	the number of MSM social network neighbours including stable sexual partners, casual sexual partners and other MSM community friends of key influential index k
M	the total number of all HIVST kits for allocation this time
variables	
$x_{k}$	the number of HIVST kits allocating to key influential index k

Objective: maximize the sum of total health economic benefit of HIVST secondary distribution for all key influential indexes,

3.1 $$\max\sum_{k\in K}\sum_{a\in A}\lfloor {\text{Pr}}^{a,k}x_{k}\rfloor E^{a},$$

where ${\text{Pr}}^{a,k}\in Q$ as well as ${\text{Pr}}^{a,k}\in [0,1]$. Here, the item $\left\lfloor {\text{Pr}}^{a,k}x_{k}\right\rfloor$ means the greatest integer number not exceeding ${\text{Pr}}^{a,k}x_{k}$ (i.e. the Gaussian greatest integer function). For example, if ${\text{Pr}}^{a,k}=0.25$ and $x_{k}=7$, then ${\text{Pr}}^{a,k}x_{k}=1.75$, thus $\lfloor {\text{Pr}}^{a,k}x_{k}\rfloor =\lfloor 1.75\rfloor =1$. The adoption of Gaussian greatest integer function here is due to the fact that we need to ensure that the output number of each type of alters is also an integer. Then we introduce constraints.

Subject to: constraints of integer decision variables, of limited resources settings, and of upper bound of kits’ number for each index can distribute.

3.2 $$\begin{array}{r} \sum_{k\in K}x_{k}\leq M \end{array}$$

3.3 $$\begin{array}{r} \forall \;k\in K,x_{k}\leq S_{k} \end{array}$$

3.4 $$\begin{array}{r} \;\text{and}\;\forall \;k\in K,x_{k}\in N^{*}. \end{array}$$

To be specific, constraints (3.2) are our kernel constraints that ensure a fixed resource setting for the optimal allocation of HIV prevention interventions.

Constraints (3.3) consider the upper bound of the number of HIVST kits that each key influential index k can distribute out in the secondary distribution program. Generally, an index cannot distribute more kits than the corresponding number of members within his social network. We already counted the number of stable sexual partners and the number of casual sex partners each index had in the past three months. We also counted the number of MSM community friends each index could contact in the next three months as part of our survey data.

Constraints (3.4) ensure that each decision variable, the number of HIVST kits allocating to key influential index k, is a non-negative integer.

### (b) Model II

Model II determined the allocation of HIV test kits to all indexes (both key influencers and non-key-influencer) who signed up for the secondary HIVST distribution program. Key influencers were considered indexes more likely to distribute more than one kit (i.e. greater than or equal to 2 kits), and thus we set one more constraint that we are supposed to allocate at least two kits to such key influencers. Additionally, the workload of the gay-led community-based organization had already increased as we considered all indexes. Hence, we primarily examined the indexes from the same residence city or the same residence province (i.e. other cities within the same province) with the gay-led community organization. It conveniently enabled participant follow-up for feedback and healthcare management post-self-testing. In practice, this strategy could expand HIV testing coverage, although this may increase the workload of healthcare service providers or social workers.

Model II was used for determining how many kits should be allocated to each index no matter whether this index is a key influential one or not, with the same final goal of maximizing the economic benefits, which considers the overall effect of normal alters expansion and positive & newly tested alters detection. We define the sets, parameters and variables of Model II in table 2. These settings in Model II are also data-driven from our actual implementation experience of an HIVST secondary distribution program. Then the Model II is defined as follows.

**Table 2.** Model II notations.

*sets*	
K	set of key influential indexes
I	set of non-key indexes
L	set of locations of indexes
A	set of types of alters
parameters	
k	indices of key influential indexes
i	indices of non-key indexes
l	indices of locations of indexes
a	indices of types of alters
c	the number of indexes of l=1 (i.e. from the same residence city with our organization)
p	the number of indexes of l=2 (i.e. from other cities within the same province)
o	the number of indexes of l=3 (i.e. from other provinces)
${\text{Pr}}_{l}^{a,k}$	probability of key influential index k at location l distributing an HIVST kit to an alter of type a
${\text{Pr}}_{l}^{i,k}$	probability of non-key index i at location l distributing an HIVST kit to an alter of type a
$E^{a}$	economic benefit of distributing an HIVST kit to an alter of type a
$S_{k,l}$	the number of MSM social network neighbours including stable sexual partners, casual sexual partners and other MSM community friends of key influential index k at location l
$S_{i,l}$	the number of MSM social network neighbours including stable sexual partners, casual sexual partners and other MSM community friends of non-key index i at location l
M	the total number of all HIVST kits for allocation this time
variables	
$x_{k,l}$	the number of HIVST kits allocating to key influential index k at location l
$x_{i,l}$	the number of HIVST kits allocating to non-key index i at location l

Objective: maximize the sum of total health economic benefit of HIVST secondary distribution for both key influential indexes and non-key indexes,

3.5 $$\max\sum_{k\in K}\sum_{a\in A}\sum_{l\in L}\lfloor {\text{Pr}}_{l}^{a,k}x_{k,l}\rfloor E^{a}+\sum_{i\in I}\sum_{a\in A}\sum_{l\in L}\lfloor {\text{Pr}}_{l}^{i,k}x_{i,l}\rfloor E^{a},$$

where ${\text{Pr}}_{l}^{a,k}\in Q$, ${\text{Pr}}_{l}^{i,k}\in Q$, as well as ${\text{Pr}}_{l}^{a,k}\in [0,1]$, ${\text{Pr}}_{l}^{i,k}\in [0,1]$. Here the item $\left\lfloor {\text{Pr}}_{l}^{a,k}x_{k,l}\right\rfloor$ means the greatest integer number not exceeding ${\text{Pr}}_{l}^{a,k}x_{k,l}$, which is the same function with Model I (i.e. the Gaussian greatest integer function). For example, if ${\text{Pr}}_{l}^{a,k}=0.8$ and $x_{k,l}=6$, then ${\text{Pr}}_{l}^{a,k}x_{k,l}=4.8$, thus $\lfloor {\text{Pr}}_{l}^{a,k}x_{k,l}\rfloor =\lfloor 4.8\rfloor =4$.

So does the item $\left\lfloor {\text{Pr}}_{l}^{i,k}x_{i,l}\right\rfloor$. The adoption of Gaussian greatest integer function here is also due to the fact that we need to ensure the output number of each type of alters is also an integer. Then we introduce constraints.

Subject to: constraints of integer decision variables, of limited resources settings, of upper or lower bound of kits’ number for each index can distribute, and of location consideration

3.6 $$\begin{array}{r} \sum_{k\in K}\sum_{l\in L}x_{k,l}+\sum_{i\in I}\sum_{l\in L}x_{i,l}\leq M \end{array}$$

3.7 $$\begin{array}{r} \frac{\left(\sum_{k\in K}+\sum_{i\in I})\sum_{l=3}(x_{k,l}+x_{i,l}\right)}{o}\leq \frac{\left(\sum_{k\in K}+\sum_{i\in I})\sum_{l=2}(x_{k,l}+x_{i,l}\right)}{p} \end{array}$$

3.8 $$\begin{array}{r} \frac{\left(\sum_{k\in K}+\sum_{i\in I})\sum_{l=2}(x_{k,l}+x_{i,l}\right)}{p}\leq \frac{\left(\sum_{k\in K}+\sum_{i\in I})\sum_{l=1}(x_{k,l}+x_{i,l}\right)}{c} \end{array}$$

3.9 $$\begin{array}{r} \forall \;k\in K,\forall \;l\in L,x_{k,l}\leq S_{k,l} \end{array}$$

3.10 $$\begin{array}{r} \forall \;i\in I,\forall \;l\in L,x_{i,l}\leq S_{i,l} \end{array}$$

3.11 $$\begin{array}{r} \forall \;k\in K,\forall \;l\in L,x_{k,l}\geq 2 \end{array}$$

3.12 $$\begin{array}{r} \forall \;k\in K,\forall \;l\in L,x_{k,l}\in N^{*} \end{array}$$

3.13 $$\begin{array}{r} \;\text{and}\;\forall \;i\in I,\forall \;l\in L,x_{i,l}\in N^{*}. \end{array}$$

To be specific, constraints (3.6) are the motivation of our study, that is, to consider a limited resource situation of HIVST secondary distribution kits’ optimal allocation for indexes.

Constraints (3.7) and (3.8) both consider the location issues. As we mentioned before, Model II increases the workload of healthcare service providers (i.e. a gay-led community organization in our program). Therefore, it would be better to decrease the workload like follow-up testing feedback collection, health management, and other similar things. It is more convenient for social workers in our gay-led community organization to operate the follow-up testing feedback and health management of indexes who come from the same city (i.e. location 1) with our organization or from other cities within the same province (i.e.location 2). As a result, constraints (3.7) and (3.8) are set, obeying the suggestions by social workers in our practical HIVST secondary distribution implementation.

Constraints (3.9) consider the upper bound of the number of HIVST kits that each key influential index k can distribute out in the secondary distribution program. Generally, an index cannot distribute more test kits than the corresponding number of members within his social network neighbours. We have already counted his stable sexual partners in the latest three months, his casual sex partners in the latest three months, and his other MSM community friends whom he could contact in the forthcoming three months, according to our survey data.

Constraints (3.10) consider the upper bound of the number of HIVST kits that each non-key index i can distribute out in the secondary distribution program. The explanations of this upper bound are similar to constraint (3.9).

Constraints (3.11) set the lower bound of the number of HIVST kits that each key influential index k is supposed to distribute out in the secondary distribution program. It is related to our previous study of identifying vital influencers via an ensemble machine learning approach [24], in which we defined key influencers as indexes that could distribute at least two kits. In other words, we set whether ‘≥2 kits’ or not for training in that machine learning classification task such constraint was hence developed.

Constraints (3.12) ensure that each decision variable for key influential index k, i.e. the number of HIVST kits allocated to every one, is a non-negative integer.

Constraints (3.13) ensure that each decision variable for non-key index i, i.e. the number of HIVST kits allocated to every one, is a non-negative integer.

## 4. Greedy algorithm

There are no existing solvers for Model I and Model II, as we incorporate the Gaussian greatest integer function in objective functions. However, if we change the objective function by temporally excluding the Gaussian greatest integer function, such as changing objective function (3.1) to

4.1 $$\sum_{k\in K}\sum_{a\in A}{\text{Pr}}^{a,k}x_{k}E^{a},$$

and changing objective function (3.5) to

4.2 $$\sum_{k\in K}\sum_{a\in A}\sum_{l\in L}{\text{Pr}}_{l}^{a,k}x_{k,l}E^{a}+\sum_{i\in I}\sum_{a\in A}\sum_{l\in L}{\text{Pr}}_{l}^{i,k}x_{i,l}E^{a},$$

then our two models both become the standard linear integer programming models. We could use existing solvers for integer programming such as CPLEX or some packages in python or R.

Assuming that we have obtained the temporary solutions from solvers in terms of objective function (4.1) and (4.2) (i.e. the temporary values of $x_{k}$ for Model I, as well of $x_{k,l}$ and $x_{i,l}$ for Model II), then we can calculate the value of (3.1) and (3.5) based on such temporary solutions, which we call temporary solver solutions.

According to the property of Gaussian greatest integer function, it holds that

4.3 $$\sum_{k\in K}\sum_{a\in A}\lfloor {\text{Pr}}^{a,k}x_{k}\rfloor E^{a}\leq \sum_{k\in K}\sum_{a\in A}{\text{Pr}}^{a,k}x_{k}E^{a},$$

and it also holds that there is a similar inequality between (3.5) and (4.2). These two inequalities tell us that the optimal value of (3.1) and (3.5) will definitely be less than or equal to the optimal value of (4.1) and (4.2), respectively. However, the temporary solver solutions-based temporary value of (3.1) and (3.5) might be further improved.

Therefore, our greedy algorithm is to fine-tune the temporary solver solutions with the final goal of getting a greater value of objective function (3.1) and (3.5), comparing with the temporary value of (3.1) and (3.5) based on the temporary solver solutions.

### (a) Algorithm development

We simplify (4.1) and (4.2), and regard them in a uniform way like

4.4 $$w_{1}x_{1}+w_{2}x_{2}+w_{3}x_{3}+\cdots +w_{n}x_{n},$$

where $w_{j}$ represents the product of the corresponding ${\text{Pr}}_{j}$ and the corresponding $E_{j}$ in terms of $x_{j}$, j=1,2,…,n, that is

4.5 $$w_{j}={\text{Pr}}_{j}E_{j}.$$

Thus, (3.1) and (3.5) could also have been rewritten in a uniform way like

4.6 $$\lfloor {\text{Pr}}_{1}x_{1}\rfloor E_{1}+\lfloor {\text{Pr}}_{2}x_{2}\rfloor E_{2}+\cdots +\lfloor {\text{Pr}}_{n}x_{n}\rfloor E_{n}.$$

Then $w_{1},w_{2},\ldots ,w_{n}$ are sequenced by the following order

4.7 $$w_{\left(1\right)}\leq w_{\left(2\right)}\leq w_{\left(3\right)}\leq \cdots \leq w_{\left(n\right)},$$

with corresponding variables $x_{\left(1\right)},x_{\left(2\right)},\ldots ,x_{\left(n\right)}$.

Assume that $x_{\left(j\right)}^{*}$ is the temporary solution of $x_{\left(j\right)}$ to maximize the value of (4.4) with linear constraints (3.2) to (3.4) or (3.6) to (3.13) by the solver. According to the property of Gaussian greatest integer function like (4.3), it holds that the value of (4.6) by $x_{\left(j\right)}^{*}$ is less than or equal to the value of (4.4) by $x_{\left(j\right)}^{*}$. Thus, we aim to increase the temporary value of (4.6) by $x_{\left(j\right)}^{*}$ while there is an upper bound for such addition.

We fine-tune $x_{\left(j\right)}^{*}$ by the rule that we substitute $x_{\left(1\right)}^{*}+1$ with $x_{\left(1\right)}^{*}$ and substitute $x_{\left(n\right)}^{*}-1$ with $x_{\left(n\right)}^{*}$, if all linear constraints are met as well and the value of (4.6) is added through such fine-tuning, then we implement this update and repeat this step again, otherwise we will check similarly in terms of $x_{\left(2\right)}^{*}+1$ and $x_{\left(n-1\right)}^{*}-1$, and so on. We illustrate our greedy algorithms for Model I and Model II by the following algorithm 1.


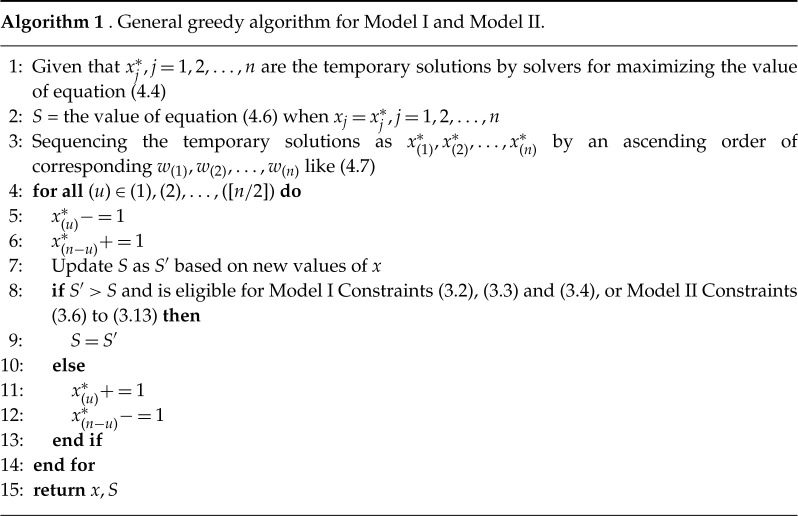


As for Model II, we further propose a sub-grouping greedy algorithm (algorithm 2) especially for Model II considering the Constraints (3.7) and (3.8). The main idea is to fine-tune x within three subgroups (i.e. indexes at three locations are classified in three subgroups). Initiating greedy algorithm in each subgroup three times then Constraints (3.7) and (3.8) automatically hold.


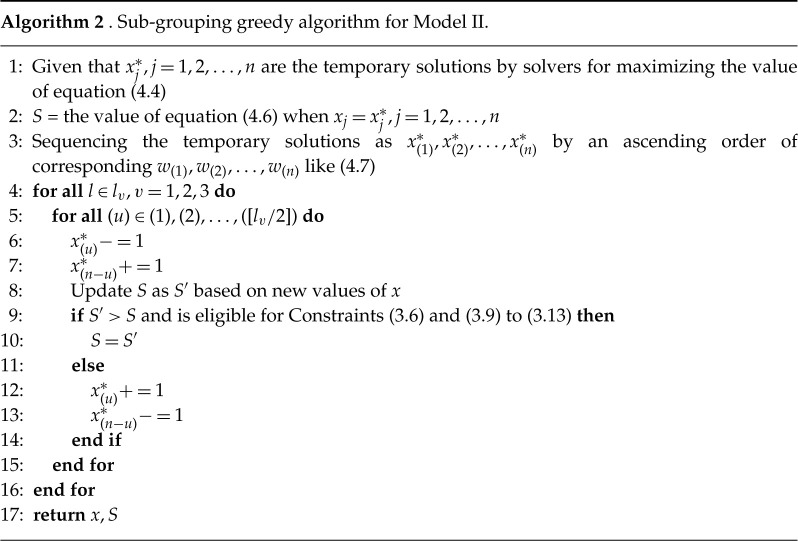


### (b) Mathematical derivation

We now explain why our greedy algorithms worked to find better solutions based on temporary solutions from solvers.

**Theorem 4.1.**

*Assume that* ${\text{Pr}}_{j}\in Q$ *and* ${\text{Pr}}_{j}\in [0,1]$, $E_{j}\in R^{+}$, $w_{j}={\text{Pr}}_{j}E_{j}$. *Consider a standard integer programming problem with objective function* $w_{1}x_{1}+w_{2}x_{2}+w_{3}x_{3}+\cdots +w_{n}x_{n}$ *with all linear constraints for non-negative integer variables* $x_{j}$. *To maximize the objective function, suppose we obtain the solutions as* $x_{j}^{*}$. *A new objective function is defined as* $\lfloor {\text{Pr}}_{1}x_{1}\rfloor E_{1}+\lfloor {\text{Pr}}_{2}x_{2}\rfloor E_{2}+\cdots +\lfloor {\text{Pr}}_{n}x_{n}\rfloor E_{n}$ *through Gaussian Greatest Integer Function. The value of the new objective function by* $x_{j}^{*}$ (*i.e*.$\lfloor {\text{Pr}}_{1}x_{1}^{*}\rfloor E_{1}+\lfloor {\text{Pr}}_{2}x_{2}^{*}\rfloor E_{2}+\cdots +\lfloor {\text{Pr}}_{n}x_{n}^{*}\rfloor E_{n}$) *is **NOT** the maximized value of the new objective function **under some conditions** within the same linear constraints*.

**Proof.**

For certain indices $j_{1}$ and $j_{2}$, the corresponding $w_{j_{1}}$ and $w_{j_{2}}$ have a certain inequality relationship. Let us say $w_{j_{1}}\leq w_{j_{2}}$, i.e. ${\text{Pr}}_{j_{1}}E_{j_{1}}\leq {\text{Pr}}_{j_{2}}E_{j_{2}}$. We now make derivations regarding under what conditions the following inequality

4.8 $$\lfloor {\text{Pr}}_{j_{1}}x_{j_{1}}^{*}\rfloor E_{j_{1}}+\lfloor {\text{Pr}}_{j_{2}}x_{j_{2}}^{*}\rfloor E_{j_{2}}\leq \lfloor {\text{Pr}}_{j_{1}}(x_{j_{1}}^{*}+1)\rfloor E_{j_{1}}+\lfloor {\text{Pr}}_{j_{2}}(x_{j_{2}}^{*}-1)\rfloor E_{j_{2}},$$

holds. As ${\text{Pr}}_{j}\in Q$ and ${\text{Pr}}_{j}\in [0,1]$, we write ${\text{Pr}}_{j_{1}}=(b_{1}/a_{1})$ where $(a_{1},b_{1})=1$ (when $b_{1}\geq 1$), $a_{1}\in N$, $a_{1}\geq 1$, $b_{1}\in N$, $b_{1}\geq 0$, $a_{1}\geq b_{1}$. Similarly, we write ${\text{Pr}}_{j_{2}}=(b_{2}/a_{2})$. Then we have $\left\lfloor {\text{Pr}}_{j_{1}}x_{j_{1}}^{*}\rfloor =\lfloor (b_{1}/a_{1})x_{j_{1}}^{*}\right\rfloor$. Assume $\lfloor (b_{1}/a_{1})x_{j_{1}}^{*}\rfloor =y_{1}$ where $y_{1}\in N$ and $y_{1}\geq 0$. Thus we have $b_{1}x_{j_{1}}^{*}=a_{1}y_{1}+r_{1}$ and the integer $r_{1}$ is the remainder of this division, i.e.$0\leq r_{1}<a_{1}$. Consider the value of $\left\lfloor (b_{1}/a_{1})(x_{j_{1}}^{*}+1)\right\rfloor$. If the equation holds that $b_{1}(x_{j_{1}}^{*}+1)=a_{1}(y_{1}+1)+r_{1}^{\prime }$ then $\lfloor (b_{1}/a_{1})(x_{j_{1}}^{*}+1)\rfloor =y_{1}+1$. The above equation requires the remainder $r_{1}^{\prime }$ of the division meets that $0\leq r_{1}^{\prime }=r_{1}+b_{1}-a_{1}<a_{1}$ and because of $a_{1}\leq 2a_{1}-b_{1}$, thus the original remainder $r_{1}$ is supposed to meet the condition that

4.9 $$a_{1}-b_{1}\leq r_{1}<a_{1}.$$

Similarly, assume $\lfloor (b_{2}/a_{2})x_{j_{2}}^{*}\rfloor =y_{2}$ where $y_{2}\in N$ and $y_{2}\geq 0$. As well $b_{2}x_{j_{2}}^{*}=a_{2}y_{2}+r_{2}$ and the integer $r_{2}$ is the remainder of this division, i.e.$0\leq r_{2}<a_{2}$. Consider the value of $\left\lfloor (b_{2}/a_{2})(x_{j_{2}}^{*}-1)\right\rfloor$. If the equation holds that $b_{2}(x_{j_{2}}^{*}-1)=a_{2}y_{2}+r_{2}^{\prime }$ then $\lfloor (b_{2}/a_{2})(x_{j_{2}}^{*}-1)\rfloor =y_{2}$ stays the same value. The above equation requires the remainder $r_{2}^{\prime }$ of the division meets that $0\leq r_{2}^{\prime }=r_{2}-b_{2}<a_{2}$, hence the original remainder $r_{2}$ is supposed to meet the condition that

4.10 $$b_{2}\leq r_{2}<a_{2}.$$

Also assume another condition that when we do $x_{j_{1}}^{*}+1$ and $x_{j_{2}}^{*}-1$, all linear constraints are satisfied. Finally, we can make a statement that under these conditions, the inequality (4.8) holds then the value of the new objective function by $x_{j}^{*}$ (i.e. $\lfloor {\text{Pr}}_{1}x_{1}^{*}\rfloor E_{1}+\lfloor {\text{Pr}}_{2}x_{2}^{*}\rfloor E_{2}+\cdots +\lfloor {\text{Pr}}_{n}x_{n}^{*}\rfloor E_{n}$) is **NOT** the maximized value of the new objective function.

## 5. Results

We compared the economic benefit of the conventional self-application method and our mathematical optimization modelling method.

First, only principally influential indexes were considered. Therefore, we compared results from their conventional self-application approach with the mathematically optimized model approach for the same index with the same limited resource M setting (i.e. a fixed number of test kits, equal to the original total number of test kits the indexes received during actual implementation by self-application). The results are depicted in table 3.

**Table 3.** Model I results.

key-influential indexes	self-application	model I solver	model I greedy algorithm
economic benefit (USD)	31 345.71	38 804.82	39 545.35

The conventional economic benefit of kits self-application was 31 345.71 USD. The temporary solutions by a solver led to a result of 38 804.82 USD. That means that the total economic benefit increased by 23% with the linear programming model. Furthermore, the greedy algorithm could raise the economic benefits of the strategy to about 39 545.35 USD. That proves the effectiveness of our algorithm.

Second, the same limited resource M was set by the same fixed total number of test kits all indexes received during actual implementation by self-application when we consider both key-influential and non-key indexes. Table 4 shows details of the results.

**Table 4.** Model II results.

all indexes	self-application	model II solver	model II greedy algorithm	model II sub-grouping greedy algorithm
economic benefit (USD)	66648.59	96424.31	96424.31	98171.57

After the subsequent addition of Model II for optimal resource allocation for all indexes, the economic benefit has increased by around 45%. Besides, in Model II, we ensure Constraints (3.7) and (3.8), which could release the workload of healthcare service providers (e.g. a gay-led community organization in our program). That includes follow-up on participants after secondary distribution and self-testing for feedback on the testing experience, participants’ health management, and so on. However, in a conventional self-application pattern, the follow-up workload of healthcare service providers is heavier.

Additionally, note that the general greedy algorithm for Model II is not as effective as for Model I, but our sub-grouping greedy algorithm plays a part. That might be owing to the fact that the fine-tuning process of general greedy algorithm is restricted by Constraints (3.7) and (3.8). The point is that in the general greedy algorithm, the fine-tuning function considers [n/2] pairs of x and over 25% pairs fail in being eligible for Constraints (3.7) and (3.8) based on our real data. In sub-grouping greedy algorithm, the fine-tuning function considers [c/2]+[p/2]+[o/2] pairs of x (where c+p+o=n). All pairs of x in the fine-tuning process of sub-grouping greedy algorithm were automatically eligible for Constraints (3.7) and (3.8). Hence, we only need to check other constraints and whether $S^{\prime }>S$. The result of the sub-grouping greedy algorithm for Model II has demonstrated this point.

## 6. Conclusion

Secondary HIVST kits distribution has proven to be an effective strategy in HIV prevention and should be upscaled in more countries [10,12]. For low-income countries (LIC) with limited healthcare resources, implementing secondary HIVST distribution might need more consideration. This study evaluated two data-driven integer programming models adopted in determining the optimal resource allocation for initial test kits dispatch in secondary HIVST distribution among Chinese MSM. Our results showed an increase in the economic benefits of secondary HIVST using the greedy algorithms developed to solve mathematical optimization problems. Such a data-driven approach to optimizing resource allocation in limited-resource settings could be used as a reference to guide the implementation of secondary HIVST distribution in LIC. Future research into this approach may lie in a quasi-experimental trial conducted from a traditional public health perspective to compare the actual economic benefit outcome of conventional self-application with that of our models and to make managerial and policy implications analysis, as this current one is a retrospective modelling study.

## Data accessibility

The data will be made available to others after obtaining the relevant data sharing agreement.
